# Thin‐Adipose Compartment at the Colonic Mesentery–Perirenal Fat Interface: Histological and Three‐Dimensional Morphological Studies

**DOI:** 10.1111/iju.70385

**Published:** 2026-03-09

**Authors:** Satoru Muro, Atsuhiko Ochi, Sho Mitsumaru, Yuki Tajika, Akimoto Nimura, Keiichi Akita

**Affiliations:** ^1^ Department of Clinical Anatomy, Graduate School of Medical and Dental Sciences Institute of Science Tokyo Tokyo Japan; ^2^ Department of Urology Kameda Medical Center Chiba Japan; ^3^ Department of Radiological Technology Gunma Prefectural College of Health Sciences Maebashi Japan; ^4^ Department of Functional Joint Anatomy, Biomedical Engineering Laboratory, Institute of Industry Incubation Institute of Science Tokyo Tokyo Japan

**Keywords:** colonic mesentery, CoMBI, retroperitoneum, surgical anatomy, three‐dimensional reconstruction

## Abstract

**Objectives:**

To elucidate the anatomical characteristics and three‐dimensional continuity of a previously unrecognized thin‐adipose compartment between the colonic mesentery and retroperitoneum, using correlative microscopy and block‐face imaging.

**Methods:**

In this study, which was conducted at the anatomical laboratory of the Institute of Science Tokyo (formerly Tokyo Medical and Dental University), seven adult cadavers were examined. Histological analysis was conducted on six specimens using paraffin sections stained with Elastica van Gieson and Masson's trichrome. One cadaver underwent three‐dimensional morphological analysis using correlative microscopy and block‐face imaging. Serial block‐face images of the perirenal region were captured at 100‐μm intervals, and three‐dimensional reconstruction segmentation was performed.

**Results:**

A distinct thin‐adipose compartment was consistently observed between the colonic mesentery and perirenal fat, enclosed by dense connective tissue and containing small vessels. Similar compartments were also found between the perirenal fat and pararenal fat, and beneath the peritoneum along the abdominal wall. These compartments extended in three directions from the peritoneal reflection and demonstrated craniocaudal continuity; laterally, these compartments converged to form a triad‐like junction.

**Conclusion:**

The thin‐adipose compartment represents a structurally organized anatomical unit rather than amorphous filler. Its consistent continuity and integration with adjacent structures support a compartment‐based framework of intra‐abdominal anatomy, which has potential relevance for understanding surgical anatomy.

## 1 Introduction

The anatomical relationship between the colonic mesentery and retroperitoneal structures has been reevaluated in parallel with advances in surgical techniques. In particular, the ascending and descending mesocolons are thought to fuse with the parietal peritoneum during fetal development, forming a fusion (Toldt's) fascia traditionally considered the boundary between the mesocolon and retroperitoneum [1]. This fascial interface is not directly penetrated by vessels or nerves. Instead, nerves traverse from the retroperitoneal side into the mesocolon, avoiding this layer [2]. Such connective tissue planes are typically avascular and used as natural dissection layers during surgery [3]. Some reports have challenged the concept of Toldt's fascia as a true fusion fascia, proposing that it represents a retracted extraperitoneal fascia formed during peritoneal remodeling [4]. Regardless of the interpretation, these connective tissue layers serve as critical anatomical interfaces between the colonic mesentery and retroperitoneum, and a precise understanding of their structure is essential for evaluating tumor invasion and ensuring safe surgical dissection.

Previous studies examining the interface between the ascending and descending mesocolons and retroperitoneal space primarily relied on histological observations from limited anatomical regions, hindering understanding of their spatial continuity and three‐dimensional architecture. Conventional anatomical approaches are insufficient to integrate macroscopic observations across extensive areas with microscopic analysis of fine structural features. To address this limitation, this study employed correlative microscopy and block‐face imaging (CoMBI), a method that enables integration of high‐resolution histological and three‐dimensional morphological analyses [5, 6, 7].

In an earlier study, we demonstrated a novel thin‐adipose compartment (TAC) in the region posterior to the kidney, and reported its anatomical relationships with the posterior renal fascia and lateral conal fascia [8]. Herein, we identified a TAC located anterior to the kidney, between the colonic mesentery and PeRF, demonstrating its anatomical relationships with the fusion fascia. We aimed to define the anatomical characteristics and spatial extent of this adipose compartment and to evaluate its continuity with adjacent structures, including the subperitoneal fat and posterior abdominal wall.

## 2 Method

### 2.1 Preparation of Cadaveric Specimens

Seven adult cadavers (six male, one female; mean age at death, 81.7 years [range, 73–92 years]) were donated to our department in accordance with the Japanese law (The Act on Body Donation for Medical and Dental Education, Act No. 56 of 1983). All donors had provided informed consent for educational and research use of their remains, which was re‐confirmed with the bereaved families. Cadavers were fixed with 8% formalin via arterial perfusion and preserved in 30% alcohol for tissue preservation. Cadavers with a history of major abdominal or pelvic surgery were excluded. Tissue blocks, including the ascending and descending mesocolons, retroperitoneum, and PeRF, were dissected en bloc.

### 2.2 Histological Analysis

Histological analysis was performed on six cadavers. In five cadavers, the entire abdomen, including the ascending and descending colons, retroperitoneum, and PeRF, was harvested en bloc, and a single transverse slice was obtained crossing both colonic mesenteries using a diamond band saw (EXAKT 312; EXAKT Technologies Inc., Germany). Representative tissue blocks containing the interface between the colonic mesentery and PeRF were excised. In the remaining cadaver, the entire abdominal region was frozen at −80°C and serially sectioned into 10‐mm‐thick transverse slices using a band saw (Nakajima Seisakusho, Y.K., Osaka, Japan), as described previously [9]. From these frozen slices, multiple smaller blocks were obtained to represent the craniocaudal extent of the adipose compartments of interest, focusing on the boundary between the colonic mesentery and retroperitoneum, and the lateral and posterior aspects of the PeRF.

All tissue blocks were fixed in 10% neutral‐buffered formalin for 24 h, dehydrated using a graded ethanol series (70%, 80%, 90%, and 100%), cleared in xylene, and embedded in paraffin under negative pressure. Serial paraffin sections (5 μm thick) were cut using a rotary microtome (RM2235; Leica Biosystems Nussloch GmbH, Wetzlar, Germany) and stained with Elastica van Gieson and Masson's trichrome staining. Microscopic examination focused on the zonal organization of adipose compartments and the distribution of small blood vessels in the mesenteric and perirenal regions.

In the current study, a TAC was defined histologically based on the following criteria: (1) a discrete adipose layer bounded on both sides by dense connective tissue lamellae; (2) clear separation from the adjacent mesenteric fat, perirenal fat (PeRF), or pararenal fat (PaRF); and (3) presence of small intracompartmental blood vessels. The present study was designed as a qualitative anatomical investigation focusing on the identification, spatial continuity, and compartmental organization of adipose tissues. Accordingly, TAC thickness measurements were provided only as approximate reference values derived from representative histological sections and were not intended as a comprehensive quantitative analysis. The formal assessment of inter‐observer reproducibility was beyond the scope of this qualitative study and should be addressed in future quantitative investigations.

### 2.3 CoMBI

One cadaver was used for three‐dimensional morphological analysis using CoMBI. The right and left perirenal regions were dissected, along with parts of the ascending and descending colons, kidneys, and abdominal walls. The specimens were fixed and embedded in opaque paraffin containing 6.25% w/w white crayon [7]. Specimen blocks were sectioned at 5 μm using a rotary‐type microtome (RX‐860; Yamato Kohki Industrial Co. Ltd., Saitama, Japan), and block‐faces were captured using a digital camera (Nikon D5100; Nikon Corporation, Tokyo, Japan) with a macro lens (Nikon AF‐S DX Micro Nikkor 40 mm *f*/2.8G; Nikon Corporation). The block‐face was captured for every 20 sections cut; 207 and 390 serial block‐face images of the right and left perirenal regions, respectively, were obtained with an interval of 100 μm. A few sections were obtained during block‐face imaging and stained with Masson's trichrome.

### 2.4 Three‐Dimensional Morphological Analysis

Segmentation and three‐dimensional reconstruction were performed using serial block‐face images obtained using CoMBI. The original three‐dimensional dataset of the left perirenal region consisted of 390 serial block‐face images with 100‐μm intervals. We created a reduced dataset of 187 block‐face images at 200‐μm intervals by selecting odd‐numbered files and excluding regions outside the area of interest. Because CoMBI acquires images directly from the cut surface, spatial alignment was inherently preserved without tissue deformation. Minor vertical misalignments were corrected manually by translation‐based alignment.

The adipose compartments and organs within the block‐face images were annotated by referring to the correlated sections stained with Masson's trichrome. The segmentation of adipose compartments and organs was performed using a combination of AI‐assisted coarse segmentation and subsequent manual refinement, as implemented with our Seg & Ref toolset (Seg & Ref; https://github.com/SatoruMuro/SAM2GUIfor3Drecon) [10]. AI assistance was used to generate an initial approximation of compartmental boundaries; thereafter, contours were manually adjusted to improve anatomical accuracy. Boundaries between the mesenteric fat, PeRF, PaRF, renal hilar fat (RHF), and TAC were defined primarily based on compartmental morphology observed in block‐face images, wherein these structures appear as distinct adipose domains separated by clear spatial interfaces. Because dense connective tissue lamellae were not directly visualized in block‐face images, correlated Masson's trichrome–stained histological sections were referenced as needed to refine anatomical boundaries.

The segments were reconstructed into a three‐dimensional surface model using 3D Slicer (version 5.2.2; https://www.slicer.org/) [11]. Three‐dimensional reconstruction was performed for the left perirenal region. Although full three‐dimensional reconstruction was not performed on the right side, corresponding block‐face images and histological sections showed comparable compartmental organization without notable left–right asymmetry.

## 3 Results

**FIGURE 1:**
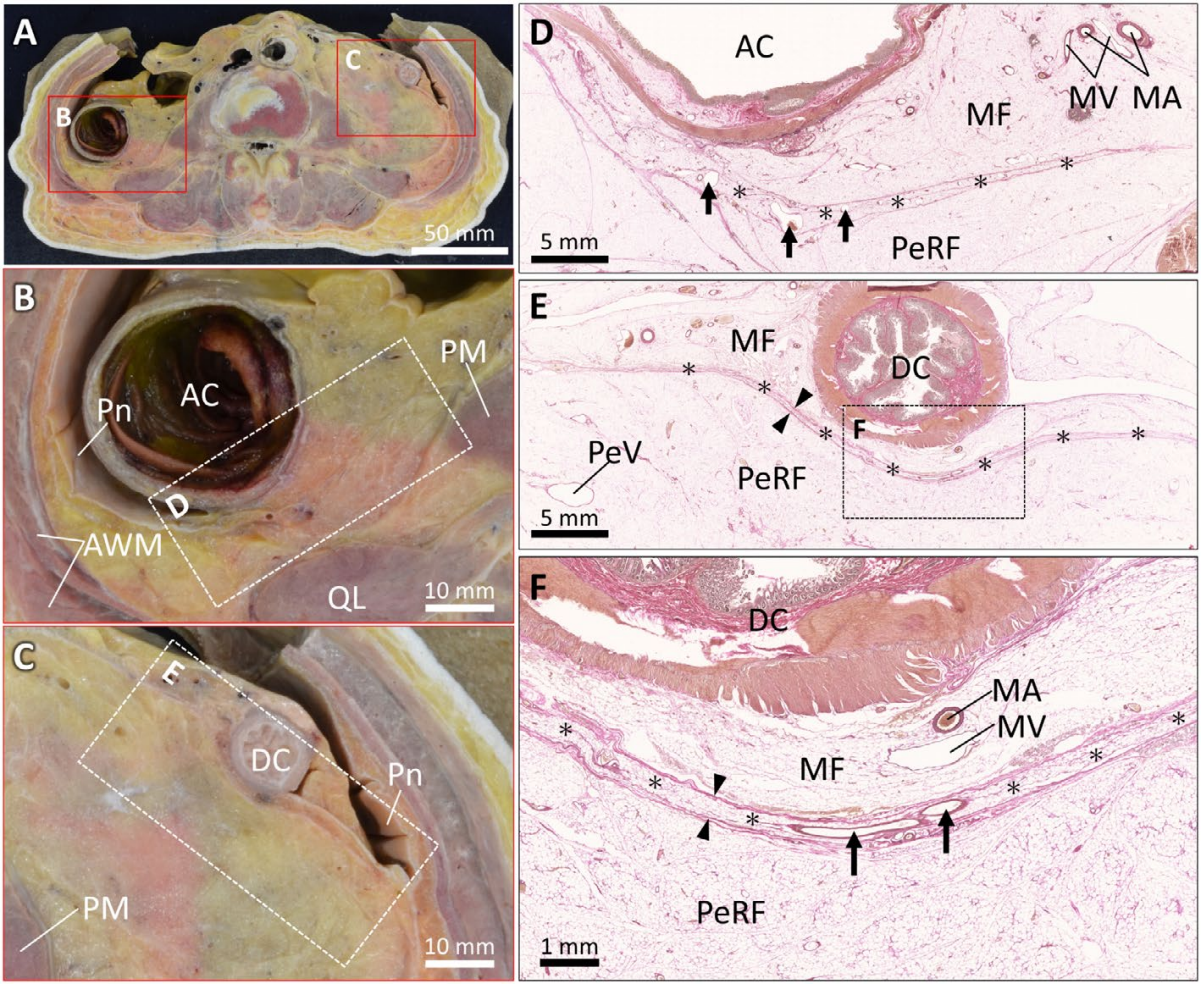
The TAC between the colonic mesentery and perirenal fat. (A) Transverse section of the abdomen showing the anatomical position of the ascending and descending colon. (B, C) Magnified view of the red rectangular frames in A, showing the peritoneal reflections at the colonic margins and their continuation to the abdominal wall. (D, E) Histological images (Elastica van Gieson staining) of the rectangular areas in B and C, showing a TAC between the MF and PeRF. The TAC is identified as a discrete adipose layer (asterisks) bound anteriorly and posteriorly by dense connective tissue lamellae (arrowheads). (F) Higher‐magnification view of the boxed area in E, highlighting small intracompartmental blood vessels (arrows) within the TAC. AC, ascending colon; AWM, abdominal wall muscle; DC, descending colon; MA, mesenteric artery; MF, mesenteric fat; MV, mesenteric vein; PeRF, perirenal fat; PM, psoas muscle; Pn, peritoneum; QL, quadratus lumborum muscle; TAC, thin‐adipose compartment.

We identified a thin compartment of adipose tissues between the ascending and descending colonic mesenteries and PeRF and named it the TAC. The colon was clearly identified in the transverse section of the abdomen (Figure 1A). The anterior surfaces of these colonic segments were covered by the peritoneum, which folded laterally at the colonic margins and continued toward the abdominal wall (Figure 1B,C). Histological examination of the interface between the mesenteric and retroperitoneal adipose tissue revealed a TAC with an approximate thickness (approximately 0.3–1.5 mm), which is provided as a reference scale rather than as a quantitative measurement (Figure 1D,E). This compartment was enclosed anteriorly and posteriorly by dense connective tissue, forming a discrete unit distinguishable from the mesenteric and retroperitoneal fat. Small blood vessels were observed within the TAC (Figure 1F).

**FIGURE 2:**
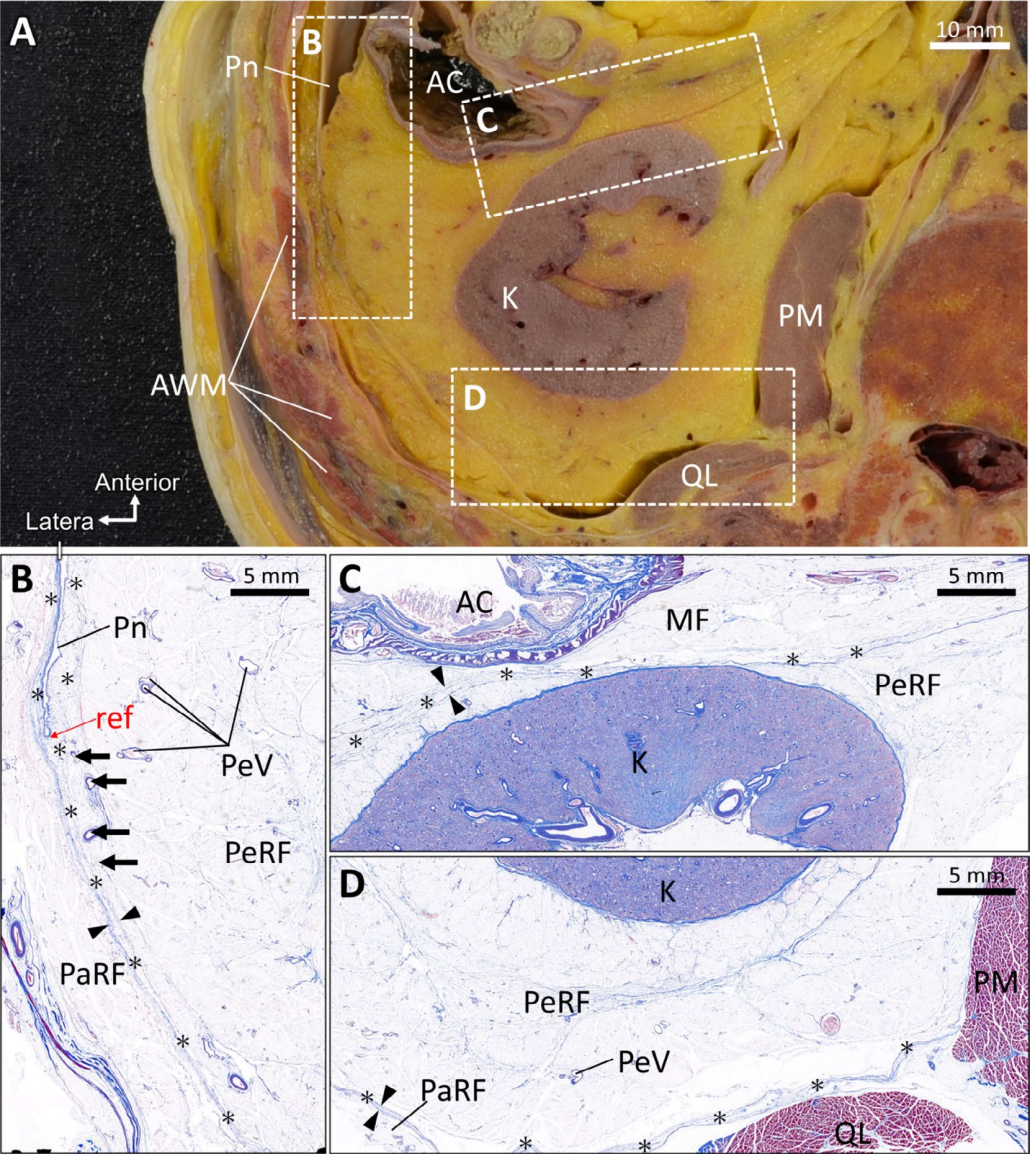
Distribution of the TAC. (A) Transverse section of the abdomen at the level of the kidney showing the tissue sampling areas in the lateral, anterior, and posterior perirenal regions. (B) Histological section (Masson's trichrome staining) of the lateral region demonstrating a TAC between the PeRF and PaRF. The TAC is identified as a discrete adipose layer (asterisks) bound by dense connective tissue lamellae (arrowheads) and containing small intracompartmental blood vessels (arrows). (C) Histological section (Masson's trichrome staining) of the anterior region showing the TAC between the MF and PeRF, identified based on the same histological criteria. (D) Histological section (Masson's trichrome staining) of the posterior region between the kidney and body wall, demonstrating a TAC located posterior to the PeRF. AC, ascending colon; AWM, abdominal wall muscle; MF, mesenteric fat; PaRF, pararenal fat; PeRF, perirenal fat; PeV, perinephric vein; PM, psoas muscle; Pn, peritoneum; QL, quadratus lumborum muscle; TAC, thin‐adipose compartment.

The presence of the TAC was confirmed at different transverse levels, including the kidney. Tissue samples were collected from the lateral, anterior, and posterior areas of the perirenal adipose tissue (Figure 2A). Histological analysis was performed in the lateral area near the colon and peritoneal reflection (Figure 2B), anterior area including the interface between the mesenteric and retroperitoneal adipose tissue (Figure 2C), and posterior area between the kidney and body wall (Figure 2D). In the lateral area, a TAC was observed between the PeRF and PaRF, with an approximate thickness (approximately 0.3–2 mm), shown here for reference. Perinephric veins were present within the PeRF, and small vessels were noted within the TAC (Figure 2B). The TAC extended from the point of peritoneal reflection in three directions: (1) anteriorly beneath the peritoneum along the body wall, (2) anteriorly between the peritoneum and PeRF, and (3) posteriorly between the PaRF and PeRF. The posterior component of the TAC appeared to be thinner than those of TACs observed in the lateral and anterior regions, with an approximate thickness (approximately 0.3–1 mm) shown for reference.

**FIGURE 3:**
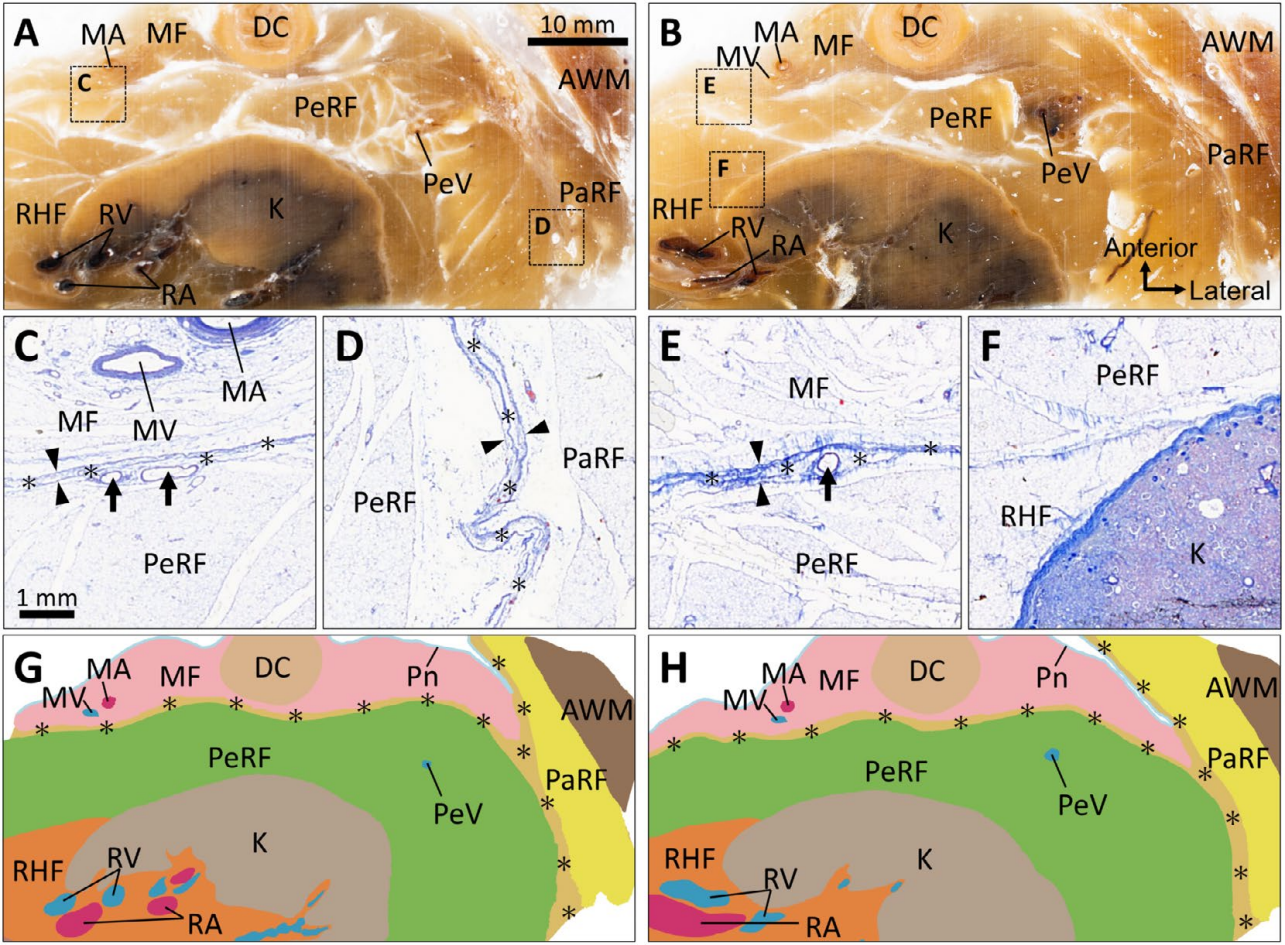
Wide‐area visualization of adipose compartments using the correlative microscopy and block‐face imaging method. (A, B) Block‐face images from large tissue specimens containing the colon, kidney, and abdominal wall, identifying the MF, PeRF, RHF, and PaRF. (C–E) Histological sections (Masson's trichrome staining) of the square frames in A and B, confirming the presence of TACs identified as discrete adipose layers (asterisks) bound by dense connective tissue lamellae (arrowheads) and containing small intracompartmental blood vessels (arrows). TACs are observed between the MF and PeRF (C, E) and between PeRF and PaRF (D). (F) Histological section (Masson's trichrome staining) demonstrating a dense connective tissue boundary separating the PeRF and RHF. (G, H) Traced and segmented images of A and B, illustrating the lateral convergence of the TACs and vertical craniocaudal continuity, forming a triad‐like anatomical junction. AWM, abdominal wall muscle; DC, descending colon; MA, mesenteric artery; MF, mesenteric fat; MV, mesenteric vein; PaRF, pararenal fat; PeRF, perirenal fat; PeV, perinephric vein; Pn, peritoneum; RA, renal artery; RHF, renal hilar fat; RV, renal vein; TAC, thin‐adipose compartment.

In the block‐face images obtained using CoMBI from a large tissue specimen, including the colon, kidney, and abdominal wall, distinct adipose compartments were identified: mesenteric fat, PeRF, RHF, and PaRF (Figure 3A,B). Cross‐sectional profiles of the blood vessels were observed within each adipose compartment. Histological sections prepared from the same specimen confirmed the presence of TACs between the mesentery and PeRF, and between the PeRF and PaRF (Figure 3C–E). The TACs were enclosed on both sides by dense connective tissue and contained small blood vessels, consistent with previous observations (Figures 1 and 2). A clear boundary composed of dense connective tissues was observed between the PeRF and RHF, indicating that these are separate compartments (Figure 3F). Wide‐field block‐face imaging obtained using CoMBI showed that TACs were consistently present (1) between the colonic mesentery and PeRF, (2) between the PeRF and PaRF, and (3) beneath the peritoneum along the abdominal wall. These compartments converged laterally with the PeRF, forming a triad‐like junction (Figure 3A–H). Serial block‐face images demonstrated that these compartments extended vertically, suggesting a continuous distribution along the craniocaudal axis. Consistent morphological features were observed in the specimens from the right perirenal region.

**FIGURE 4:**
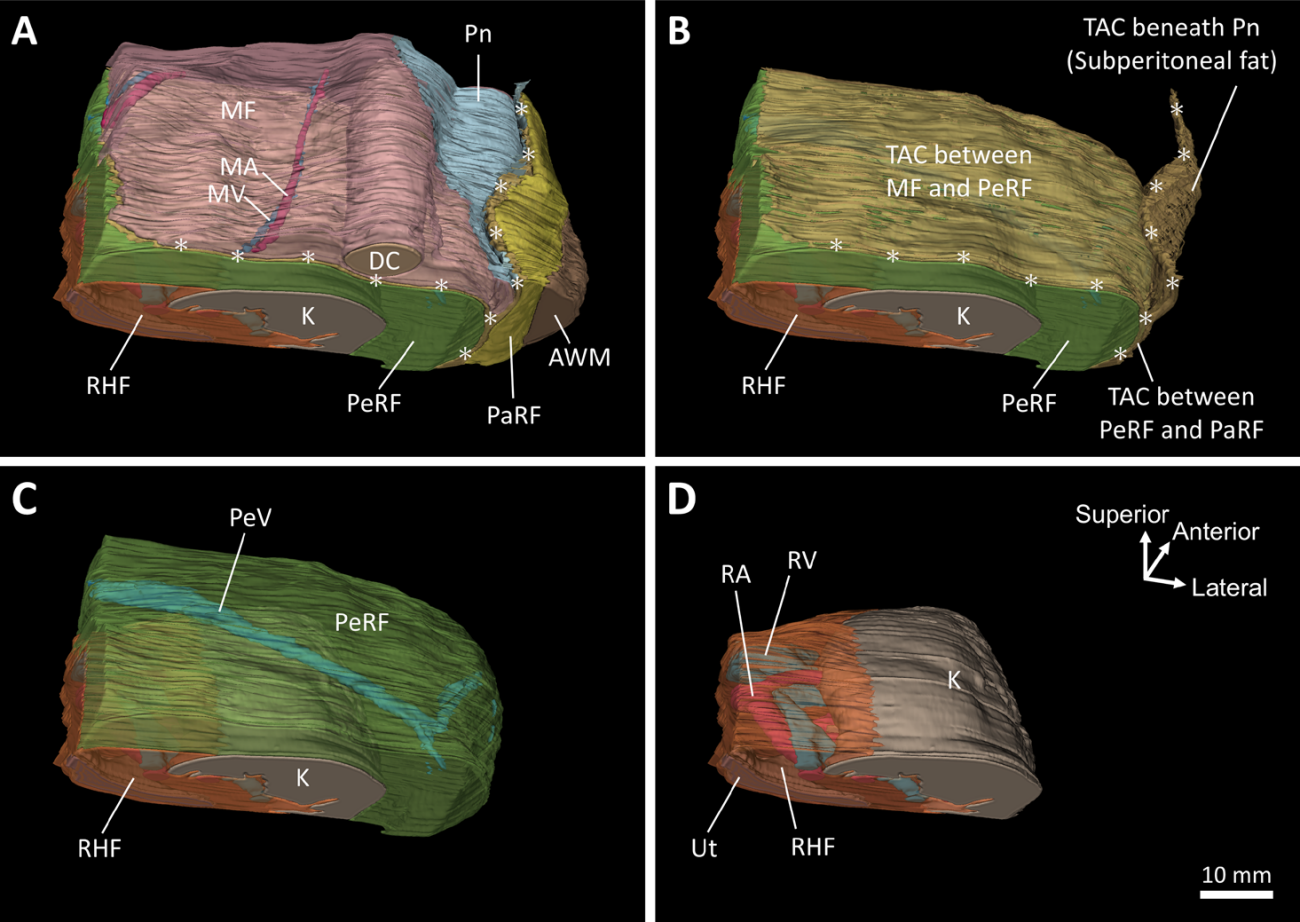
Three‐dimensional reconstruction of the adipose compartments and associated organs. (A) The descending colon surrounded by the MF and peritoneal fold continuing toward the abdominal wall. (B) TACs located posterior to the mesentery and subperitoneally along the lateral abdominal wall, extending toward the compartment between the PeRF and PaRF. (C) PeRF surrounding the kidney and containing PeVs. (D) RHF enclosing the renal artery, vein, and ureter. The orientation shown in panel D applies to panels A–C. AWM, abdominal wall muscle; DC, descending colon; K, kidney; MA, mesenteric artery; MF, mesenteric fat; MV, mesenteric vein; PaRF, pararenal fat; PeRF, perirenal fat; PeV, perinephric vein; Pn, peritoneum; RA, renal artery; RHF, renal hilar fat; RV, renal vein; TAC, thin‐adipose compartment; Ut, ureter.

To reveal the three‐dimensional distribution of the adipose compartments, we created segments of the adipose compartments and organs in serial block‐face images and reconstructed them into three‐dimensional images (Figure 4). The descending colon was enveloped by the mesenteric fat, which contained the vascular structures that supply the intestine. The anterior surface of the mesenteric fat was covered by the peritoneum, which folded laterally and continued toward the abdominal wall (Figure 4A). TACs were identified posterior to the mesenteric fat and beneath the peritoneum along the abdominal wall, which converged laterally at the outer aspect of the PeRF and extended posteriorly toward the compartment located between the PeRF and PaRF (Figure 4B). The PeRF, which enveloped the kidney, contained the perinephric vein (Figure 4C). The RHF enclosed the renal artery, vein, and ureter, and covered the renal hilum (Figure 4D).

## 4 Discussion

**FIGURE 5:**
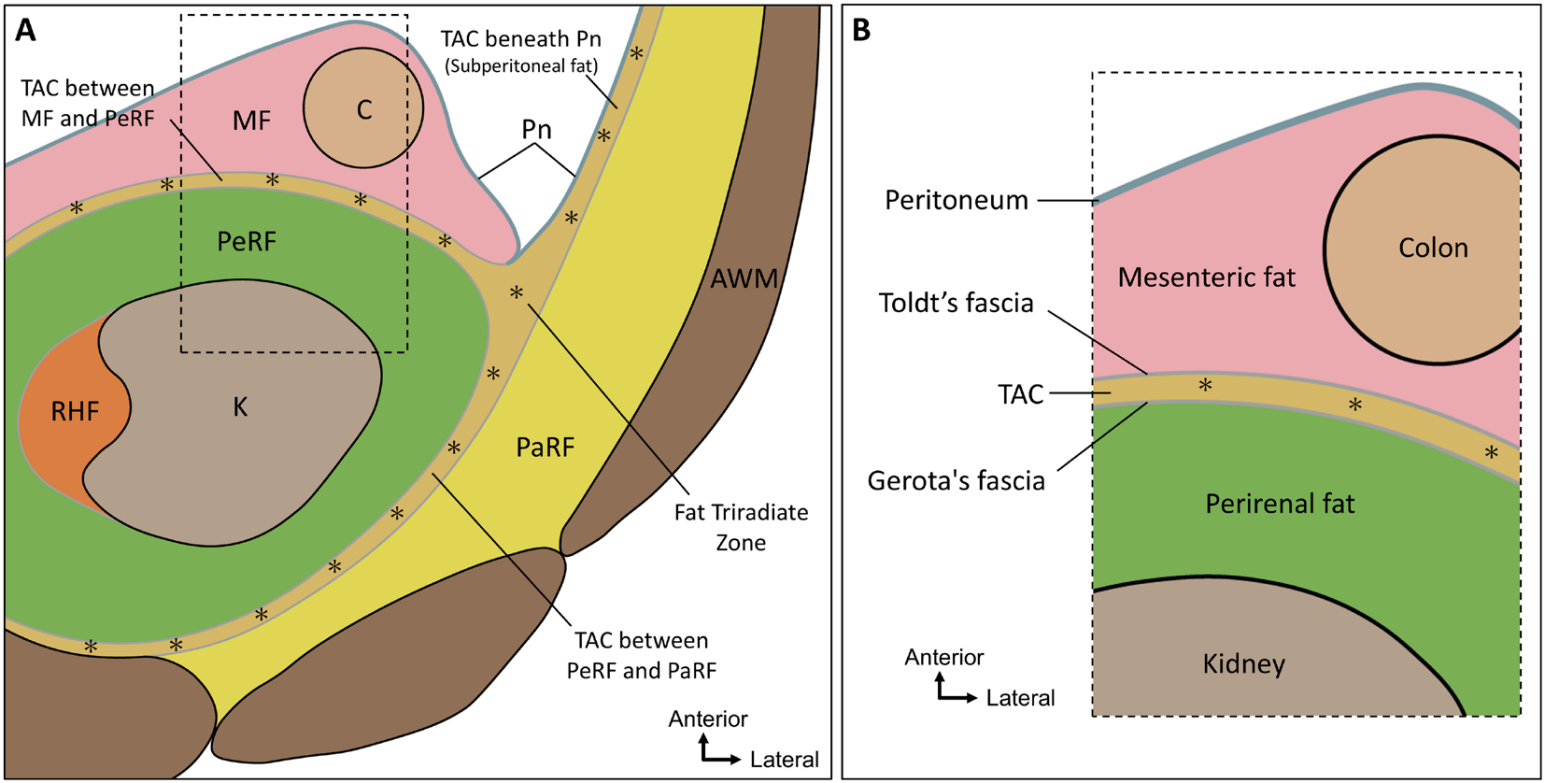
Spatial distribution and continuity of TACs revealed using histological and three‐dimensional imaging. (A) Schematic summary illustrating the anatomical continuity of the TACs identified in this study. These compartments are located at the interface between the colonic mesentery and PeRF, extending both to subperitoneal fat of the abdominal wall and posteriorly between the PeRF and PaRF (asterisks). The TACs converge to form a triad‐like junction (fat triradiate zone) lateral to the PeRF. This spatially organized structure, enclosed by dense connective tissue and containing vascular elements, suggests a spatially organized anatomical unit that may be relevant to surgical dissection. (B) Boundary structures between the colonic mesentery and the retroperitoneum. The TAC exists between the mesenteric and perirenal fat. Dense connective tissue is interposed at the boundaries between these different fat compartments. The dense connective tissue between the mesenteric fat and the TAC may correspond to what has historically been described as Toldt's fascia, whereas the tissue between the TAC and the perirenal fat may correspond to Gerota's fascia. This panel represents an interpretative anatomical model based on spatial relationships observed in the present study. Orientation arrows are shown in each panel. AWM, abdominal wall muscle; C, colon; K, kidney; MF, mesenteric fat; PaRF, pararenal fat; PeRF, perirenal fat; PeV, perinephric vein; Pn, peritoneum; RHF, renal hilar fat; TAC, thin adipose compartment.

Herein, a TAC was identified between the colonic mesentery and PeRF. It extended beyond the mesentery–retroperitoneum interface to connect with similar structures located subperitoneally and along the lateral and posterior margins of the PeRF (Figure 5A). When integrated with our previous observations of a posterior TAC [8], these findings indicate continuity of anterior and posterior TACs around the PeRF, extending laterally toward the anterolateral abdominal wall. The anatomical distinction between the PeRF and RHF was established in our previous study [12]. Accordingly, the present study does not describe a separate adipose layer but provides anatomical evidence for the anterior component of a continuous TAC system forming an organized interface at the mesentery–retroperitoneum boundary. Using three‐dimensional block‐face imaging and histology, this compartment exhibited craniocaudal continuity, indicating a vertically organized adipose morphology with potential structural, functional, and surgical relevance. From a surgical perspective, this architecture may help explain variability in the depth and character of the dissection plane between the colon and kidney. In urologic procedures, including open, laparoscopic, and robot‐assisted nephrectomy, the presence of a continuous TAC may underlie relatively avascular dissection planes, whereas the lateral “fat triradiate zone” may represent an area requiring careful intraoperative interpretation.

The conventional anatomical understanding of the relationship between the colonic mesentery and PeRF has centered on fusion fasciae, such as Toldt's and Fredet's fasciae, believed to arise from embryological peritoneal reflections [13, 14, 15, 16]. However, consistent histological identification of such fasciae remains elusive. Herein, we identified a TAC between the colonic mesentery and PeRF, clearly delineated by dense connective tissue containing small vessels on both sides, forming a distinct anatomical unit. Although similar structures have been previously described, they were often regarded as amorphous fat or poorly defined transition layers [1, 3, 17, 18, 19]. Anatomical interpretations of intra‐abdominal connective tissue and fat can be divided into two frameworks: one highlighting fascia‐like membranes as dissection planes [4], and the other treating organs, vessels, and surrounding adipose tissue as integrated compartments [8, 12]. Based on the latter view, our findings challenge the fascia‐centered model and support adipose compartments as structured, functionally relevant units.

The boundary structures between the colonic mesentery and retroperitoneum, particularly Toldt's and Gerota's fasciae, have long attracted surgical attention [13, 20]. Toldt's fascia has been described as a connective tissue plane at the interface between the mesocolic fat and retroperitoneal space, although its precise anatomical and histological correlates remain debated [13]. Some studies have interpreted thin adipose layers between the colonic mesentery and PeRF—corresponding to the layer described as the TAC in the present study—as Toldt's fascia [3, 17, 18, 19]; however, such interpretations have often been based on limited anatomical regions. Gerota proposed that the renal fascia consists of anterior and posterior layers enclosing the kidney together with the PeRF [21]; however, multiple perspectives exist regarding the fascia enveloping the kidneys and great vessels [20, 22], and its relationship with Toldt's fascia remains unclear [23]. Figure 5B presents the compartmental structure of fat at the colonic mesentery–PeRF interface. If the dense connective tissue located between the mesenteric fat and the TAC is interpreted in light of classical anatomical descriptions, it may correspond to what has historically been described as Toldt's fascia, while the dense connective tissue between the TAC and the PeRF may correspond to Gerota's fascia. This interpretation should be regarded as an anatomical mapping based on spatial relationships rather than as a definitive one‐to‐one identification of eponymous fasciae. A clearer understanding of the TAC may contribute to elucidating the layered anatomical architecture of this interface.

As demonstrated herein, the fat layer between the peritoneum and abdominal musculature in the abdominal wall is divided into two compartments (Figure 5): a TAC directly beneath the peritoneum, and a thicker compartment adjacent to the abdominal muscles. Based on their configuration and spatial relationship to the PeRF, we suggest that the urogenital structures—including the mesonephros, metanephros, and adrenal glands—originate within the TAC. The formation of mesonephric ridges in the retroperitoneum and the derivation of the adrenal cortex from the coelomic epithelium [24, 25] are consistent with the interpretation that these structures arise within the TAC beneath the peritoneum. The finding that the fat in the abdominal wall and retroperitoneum is not homogeneous but organized into discrete compartments likely reflects the spatial patterning of developmental processes.

Traditionally, fasciae have been regarded as critical surgical landmarks defining dissection planes. By contrast, the significance of adipose tissue and its surrounding connective structures as anatomical planes of separation has been highlighted [3, 4]. When considering these adipose‐based planes, accounting for inter‐individual variability—particularly in adipose tissue volume—is important. Given how thin the adipose compartment described in the present study is, its visibility may vary with individual adiposity. Adipose tissue is a passive energy store and active endocrine organ with considerable inter‐individual variability; its distribution differs by anatomical region [26, 27]. Accordingly, in individuals with minimal adipose volume, the dense connective tissue layers of Toldt's and Gerota's fasciae may lie in close proximity or fuse, causing the colonic mesenteric fat and PeRF to appear directly apposed by a single connective tissue sheet. In such cases, the dissection plane may become indistinct and the layer recognized as Toldt's fascia may shift depending on the operator's interpretation. Boekestijn et al. suggested that retroperitoneal fascial structures may function as potential spaces whose recognition is influenced by surgical manipulation and surrounding fat volume [28]. Such considerations likely apply to the anterior region of the kidney, involving Toldt's and Gerota's fasciae, and the posterior region, involving Gerota's fascia and the lateral conal fascia [8]. Although the TAC itself may not be directly visualized on routine cross‐sectional imaging, its compartmental continuity may be inferred from the characteristic distributions of retroperitoneal fat and fascial planes, as discussed in recent radiologic literature. In this context, the TAC identified herein may represent a structural substrate underlying such potential spaces, becoming apparent or obscured depending on adipose volume and surgical conditions. The “no fusion fascia” hypothesis proposed by Chen et al. [4] emphasizes that fascia‐like planes arise from continuity and reorganization of subperitoneal connective tissue rather than epithelial fusion of the peritoneum. The compartmental morphology observed herein is compatible with the no fusion fascia hypothesis but equally consistent with classical concepts based on peritoneal reflection and fusion, and thus neither confirms nor refutes this hypothesis while providing a structural framework accommodating both interpretations of retroperitoneal compartmental anatomy.

This study had some limitations. First, all specimens were obtained from formalin‐fixed elderly cadavers; aging and fixation may have influenced adipose volume and tissue visibility. Second, the sample cohort was predominantly male, which limits the assessment of sex‐related differences in adipose‐compartmental organization. Third, quantitative information on body habitus was unavailable. Adipose volume may influence the apparent configuration of the TAC, with dense connective tissue layers appearing closely apposed in lean individuals and more expanded in those with greater adiposity. Fourth, three‐dimensional reconstruction using CoMBI was performed in a single cadaver, although comparable compartmental organization was qualitatively confirmed in additional specimens. Finally, although no gross abdominal pathology or prior surgery affecting anatomical relationships was identified, subtle age‐related or subclinical changes cannot be excluded.

In conclusion, the presence of the TAC identified herein suggests that retroperitoneal adipose tissue is a spatially organized structural compartment. Accordingly, our findings offer a novel framework based on the compartmentalization and spatial distribution of adipose tissues. This approach has potential implications for improving surgical safety and precision. Although the present study did not examine pathological specimens, the continuous nature of the TAC suggests a potential anatomical route for preferential extension of inflammatory or neoplastic processes, warranting further investigation in oncologic and imaging‐based studies.

## Author Contributions

**Satoru Muro:** conceptualization, methodology, software, data curation, investigation, validation, formal analysis, supervision, visualization, project administration, resources, writing – review and editing, writing – original draft. **Atsuhiko Ochi:** conceptualization, validation, visualization, writing – review and editing. **Sho Mitsumaru:** data curation, investigation, writing – review and editing. **Yuki Tajika:** methodology, software, writing – review and editing, funding acquisition. **Akimoto Nimura:** visualization, writing – review and editing, supervision. **Keiichi Akita:** conceptualization, supervision, resources, writing – review and editing, visualization.

## Funding

This work was supported by Japan Society for the Promotion of Science (Grant 23K06299).

## Ethics Statement

The Human Subjects Research Ethics Review Committee of the Institute of Science Tokyo, Tokyo, Japan (approval number: M2019‐075). All procedures were conducted in accordance with relevant guidelines and regulations.

## Consent

All donors voluntarily agreed that their remains would be used for educational and research purposes before their death. Written informed consent was obtained from the bereaved families, and no objections were raised.

## Conflicts of Interest

The authors declare no conflicts of interest.
